# Supplementary material for: A fault-intrusion-tolerant system and deadline-aware algorithm for scheduling scientific workflow in the cloud
Source: PeerJ Comput Sci. 2021 Nov 2;7:e747. doi: 10.7717/peerj-cs.747 (PMC8576554; doi:10.7717/peerj-cs.747)
Supplement: Supplemental Information 6 [file peerj-cs-07-747-s006.zip › Myproject1.1/docs/org/cloudbus/cloudsim/examples/power/planetlab/class-use/NonPowerAware.html]

Uses of Class org.cloudbus.cloudsim.examples.power.planetlab.NonPowerAware


JavaScript is disabled on your browser.


- Overview
- Package
- Class
- Use
- Tree
- Deprecated
- Index
- Help

- Prev
- Next

- Frames
- No Frames

- All Classes

## Uses of Class org.cloudbus.cloudsim.examples.power.planetlab.NonPowerAware

No usage of org.cloudbus.cloudsim.examples.power.planetlab.NonPowerAware

- Overview
- Package
- Class
- Use
- Tree
- Deprecated
- Index
- Help

- Prev
- Next

- Frames
- No Frames

- All Classes
